# Fractionated reirradiation of recurrent high-grade gliomas: Safety with higher reirradiation dose and larger targets

**DOI:** 10.1093/noajnl/vdaf004

**Published:** 2025-01-22

**Authors:** Michael C LeCompte, Neil Vuppala, Juan M Reyes, Brandi Page, Victoria Croog, Ellen Huang, Kristin J Redmond, Lawrence R Kleinberg

**Affiliations:** Department of Radiation Oncology and Molecular Radiation Sciences, Johns Hopkins University, Baltimore, Maryland, USA; Alabama College of Osteopathic Medicine, Dothan, Alabama, USA; Department of Radiation Oncology and Molecular Radiation Sciences, Johns Hopkins University, Baltimore, Maryland, USA; Department of Radiation Oncology and Molecular Radiation Sciences, Johns Hopkins University, Baltimore, Maryland, USA; Department of Radiation Oncology and Molecular Radiation Sciences, Johns Hopkins University, Baltimore, Maryland, USA; Department of Radiation Oncology and Molecular Radiation Sciences, Johns Hopkins University, Baltimore, Maryland, USA; Department of Radiation Oncology and Molecular Radiation Sciences, Johns Hopkins University, Baltimore, Maryland, USA; Department of Radiation Oncology and Molecular Radiation Sciences, Johns Hopkins University, Baltimore, Maryland, USA

**Keywords:** glioblastoma, glioma, radiotherapy, reirradiation, toxicity

## Abstract

**Background:**

The optimal regimen, normal tissue tolerances, and appropriate indications for reirradiation for recurrent high-grade glioma (HGG) are uncertain. The aim of this study was to determine whether higher reirradiation dose was associated with toxicity or survival.

**Methods:**

Patients with HGG treated with fractionated reirradiation at a single institution from 2007 to 2022 were retrospectively reviewed. Metrics evaluated included overall survival (OS), prognostic factors for survival, and treatment-related toxicity.

**Results:**

Two hundred and thirty patients with recurrent HGG were reviewed. Median follow-up was 8.8 months. Median reirradiation dose was 41.4 Gy with 80.4% receiving concurrent systemic therapy. Median cumulative maximum doses to brainstem and optic structures were 77.9 Gy (range: 4.6–146.0 Gy) and 55.1 Gy (3.3–106.3 Gy), respectively. No injuries to these structures were identified. Radiation necrosis (RN) was identified in 9.4%. There were no significant associations between RN and target size, systemic therapy use, or reirradiation dose. Median OS was 10.2 months from reirradiation start. On multivariate analysis, improved OS was associated with better KPS, longer interval between radiotherapy sessions, reirradiation at first recurrence, and reirradiation dose ≥ 41.4 Gy. Median OS for those with IDH wildtype glioblastoma was 8.7 months. On multivariate analysis of an IDH wildtype disease subanalysis, improved OS was associated with longer interval between radiotherapy sessions and higher reirradiation dose.

**Conclusions:**

These data support the safety and efficacy of fractionated reirradiation for recurrent HGG. They suggest higher reirradiation dose may be feasible, including for large treatment volumes and for tumors near the brainstem or optic structures.

**Key Points**

- Median overall survival for all patients was 10.2 months from reirradiation start.
- Radiation necrosis was seen in 9.4% with no association with target size or reirradiation dose.
- Higher reirradiation dose (≥41.4 Gy) was associated with improved survival.

**Importance of the Study**

Practice patterns of reirradiation for recurrent high-grade gliomas (HGG) vary widely with no set standard. The current study found higher reirradiation dose for recurrent HGG to be associated with improved survival while maintaining safety. The current study includes a large proportion of patients with large treatment volumes. Previously, predictive model analyses have attempted to define cumulative dose constraints toward toxicities, including for symptomatic radiation necrosis, brainstem injury, and optic apparatus injury. This study did not find associations between treatment-related toxicities and these suggested dose constraints or with larger treatment volumes. This study supports using higher reirradiation doses for recurrent HGG and suggest there may be a survival benefit with this approach. Further study of cumulative cranial dose constraints is needed to better guide reirradiation strategies.

The management of recurrent high-grade glioma (HGG) remains a significant clinical challenge with no clear standard treatment regimen. Previous publications suggest that reirradiation, with or without concurrent systemic therapy is associated with median rates of survival ranging from 6 to 13 months after reirradiation.^1–6^ However, questions remain regarding the appropriate size of reirradiation target volumes, tumor molecular features that benefit from reirradiation, reirradiation dose/fractionation, and cumulative dose constraints to critical intracranial structures including the optic nerves/chiasm and brainstem.

Our group has regularly offered conventionally fractionated reirradiation options for those with recurrent HGG, albeit at a dose reduced from that utilized in the initial radiotherapy course. This strategy was based on a hypothesis that conventionally fractionated reirradiation may be safer than hypofractionated regimens, allow longer periods of concurrent administration of systemic therapy, and serve as a backbone toward clinical trials of new concurrent therapies. Our group does not routinely use bevacizumab (BEV). We report survival outcome and toxicity data, including the association with clinical and dosimetric characteristics, with the goal of identifying treatment factors to guide optimal implementation of this approach.

## Materials and Methods

Patients treated at our institution from 2007 to 2022 were retrospectively reviewed with approval from the institutional review board. The inclusion criteria were (1) prior treatment with conventionally fractionated radiotherapy for a glioma diagnosis WHO grade 1–4); (2) development of a recurrent or progressive WHO grade 3 or 4 glioma with or without surgical resection (initial low grade gliomas were included if pathology confirmed transition to HGG) treated at our institution with fractionated reirradiation; and (3) at least 1 follow-up visit at our institution after reirradiation.

The European Society for Radiotherapy and Oncology (ESTRO)/European Organisation for Research and Treatment of Cancer (EORTC) consensus guideline on reirradiation was used to guide the reporting of variables within this study.^7^ The following variables were collected for eligible patient at the time of repeat radiotherapy: (1) patient information (age, sex, Eastern Cooperative Oncology Group performance status, Karnofsky performance status [KPS], date of initial diagnosis based on initial pathology confirming glioma, initial glioma grade, O-6-methylgauanine-DNAmethyltransferase [MGMT] and isocitrate dehydrogenase [IDH] mutation status, histology, date of first disease recurrence by magnetic resonance imaging [MRI] or surgical pathology, date of disease recurrence before reirradiation, number of disease recurrences between initial radiotherapy and reirradiation); (2) treatment characteristics for both initial radiotherapy and reirradiation (treatment dates, technique, size of target volume, dose including maximum dose to the optic apparatus and brainstem, reirradiation field overlap, and steroid use within 3 months of treatment); (3) systemic therapy use with initial radiotherapy and reirradiation; (4) clinical outcomes (neurologic side effects, Radiation Therapy Oncology Group [RTOG] acute and late central nervous system [CNS] toxicity, radiation necrosis, and date and cause of death). Per the ESTRO/EORTC guidelines, reirradiation field overlap was defined as type 1 meaning having overlap of irradiation volumes versus type 2 meaning no overlap of irradiated volumes but with the concern for toxicity from cumulative doses.^7^

### Treatment Technique

All patients included were treated with fractionated external beam radiotherapy. Those who were treated with stereotactic radiosurgery (SRS) or stereotactic radiotherapy (SRT) were excluded. Patients were immobilized for treatment using a thermoplastic mask. Radiotherapy was delivered in 1.5 to 3.5-Gy fractions. Total does ranges stratified by most common dose per fraction were the following: 1.5 Gy/fraction (range: 30–45 Gy), 1.8 Gy/fraction (range: 30.6–59.4), 2 Gy/fraction (range: 36–60 Gy), and 3.5 Gy/fraction (all treated to 35 Gy). Dose decisions were based on the likely safe and tolerable highest dose per the treating physician. This process considered the extent of treatment field overlap, locations of critical structures, and the time between radiation treatments. The standard reirradiation gross tumor volume was the T1-weighted contrast-enhancing lesion on MRI with a 0.5 to 1.0 cm margin to clinical target volume (CTV) and a 0.5 cm margin to planning target volume (PTV). When given, temozolomide (TMZ) was typically administered at a 75 mg/m^2^ daily dose concurrently with radiotherapy. BEV was typically administered at 10 mg/kg every 14 days, when utilized.

Composite plans of the initial radiation and reirradiation treatments were used to determine cumulative dose to the optic apparatus (optic nerves, chiasm) and brainstem, when available. Otherwise, the cumulative dose to these structures was reported by adding the maximum point dose from the combined plans using the cautious assumption of complete overlap over these points. This is with the concern that the plan and image fusion technology, when anatomy may be distorted by intervening progression and treatment interventions, is not accurate enough to determine overlap of individual points with precision. The criteria varied and were determined by the treating physician with reference to institutional suggested guidelines. For the optic apparatus, a typical maximum point dose accepted in the reirradiation setting would be 24 Gy (underdosing the target if needed) if the optic nerves and chiasm had previously received a dose near the standard tolerance criteria of 50 to 54 Gy. The brainstem constraint was a cumulative maximum potential reirradiation point dose of 100 Gy. Cumulative equivalent dose in 2-Gy fraction (EQD2) were calculated for each plan. Prescriptions doses were used to calculate the EQD2 of each plan by using the linear quadratic formula and an alpha/beta of 3.

### Outcomes Analyzed and Statistical Analysis

Clinical outcomes assessed were the date and cause of death and date of last follow-up. The number of disease recurrences between radiation treatments were used to determine whether reirradiation was given at first recurrence versus subsequent recurrence. Radiation-related neurotoxicity outcomes assessed were neurologic side effects at last follow-up, RTOG acute and late CNS toxicity,^8^ dexamethasone requirement, and radiation necrosis defined by imaging or pathology.^3^ Grade of myelosuppression within 1 month of reirradiation was collected as defined by Common Terminology Criteria for Adverse Events version 5.0 for leukopenia, lymphopenia, neutropenia, anemia, and thrombocytopenia.^9^ OS was measured as time to death from start of reirradiation. Follow-up length was defined as the time from start of reirradiation to the last oncology visit (if patient was still alive) or death.

Survival was estimated using Kaplan–Meier methodology. Associations with OS were evaluated using univariate and multivariate Cox proportional hazard models. Factors significant on univariate analysis were included in the multivariate analysis except for initial glioma grade (due to correlation with time between radiation treatments), concurrent BEV use (due to selection bias), lymphopenia (due to only a proportion of patients having adequate CBC data), and IDH mutation status. A subanalysis of those with IDH wildtype disease who completed reirradiation was completed. The effect of treatment parameters on CNS toxicity and radiation necrosis were estimated using chi squared tests or Fisher’s exact test (for samples with any group having *n* < 5). Statistical analyses were completed using STATA v18.0 software (College Station, TX).

## Results

Two hundred and thirty patients were included in this study. Patient characteristics are presented in **Table 1**. At the time of initial diagnosis, most patients had a grade 3–4 glioma (82.2%, 189/230) and received concurrent TMZ with their initial radiation treatment (83.9%, 193/230). The median initial radiotherapy dose received for patients with HGG was 60 Gy (range: 45–75 Gy), while the median dose for those with a low-grade glioma was 54 Gy (range: 50.4–60 Gy). IDH mutation and MGMT promoter methylation status were unknown for many cases treated in the 2000s (28.3% [65/230] and 32.2%, [74/230], respectively). Of those with available information, 65.5% (108/165) were IDH wildtype (34.5% IDH mutated [57/165), and 49.4% (77/156) had MGMT promoter methylation (50.6% nonmethylated [79/156). Patients had a median follow-up of 8.8 months (range: 0.6–76.6 months).

**Table 1.** Patient Demographics

Characteristics	All Patients		IDH Wildtype Subanalysis	
	Number	%	Number	%
Total number of patients	230		99	
Sex				
Male	138	60.0	65	65.7
Female	92	40.0	34	34.3
Age, y	Median: 51 (range, 14–83)		Median: 54 (range, 14–83)	
KPS	Median: 80 (range 40–100)		Median: 80 (range 40–100)	
≤80	161	70.0	66	66.7
>80	63	27.4	32	32.3
Unknown	6	2.6	1	1.0
Initial grade glioma				
1–2	41	17.8	6	6.1
3	59	25.7	13	13.1
4	130	56.5	80	80.8
Initial radiotherapy dose				
	Grade 1–2 Median: 54 Gy (range: 50.4–60 Gy)		Grade 1–2 Median: 54 Gy (range: 54–60 Gy)	
	Grade 3–4 Median: 60 Gy (range: 45–75 Gy)		Grade 3–4 Median: 60 Gy (range: 50.4–75 Gy)	
Concurrent TMZ with initial RT				
Yes	193	83.9	91	91.9
No	28	12.2	5	5.1
Unknown	9	3.9	3	3.0
Recurrent grade glioma				
3	56	24.4	N/A	
4	174	75.7	N/A	
Reirradiation at First or Subsequent Recurrence				
First	134	58.2	64	64.7
Subsequent	95	41.3	35	35.4
Unknown	1	0.4	0	0
MGMT promoter				
Nonmethylated	79	34.4	54	54.5
Methylated	77	33.5	38	38.4
Unknown	74	32.2	7	7.1
IDH				
Wildtype	108	47.0	N/A	N/A
Mutated	57	24.8	N/A	N/A
Unknown	65	28.3	N/A	N/A

Abbreviations: IDH = Isocitrate dehydrogenase; y = year; KPS = Karnofsky performance status; Gy = gray; TMZ = temozolomide; RT = radiotherapy; N/A = not applicable; MGMT = O^6^-methylguanine DNA methyltransferase.

Ninety-three percent of patients (215/230) were treated with conventionally fractionated reirradiation using 1.8–2 Gy fractions. The remaining were treated using either 2.2–3.5 Gy- (5.2%, 12/230) or 1.5 Gy fractions (1.3%, 3/230). Common fractionation schemes were 45 Gy in 25 fractions (38.3%, 88/230), 36 Gy in 20 fractions (22.6%, 52/230), 41.4 Gy in 23 fractions (16.1%, 37/230), 39.6 Gy in 22 fractions (5.7%, 13/230), and 35 Gy in 10 fractions (3.0, 7/230). Most patients were treated with reirradiation at their first recurrence of disease (58.2%, 134/230) (**Table 1**). For all patients, median EQD2 prescription dose at initial RT was 60 Gy (range: 53.6–82.5 Gy), and the median EQD2 prescription dose received at reirradiation was 40.0 Gy (range: 1.7–60 Gy). The median EQD2 cumulative prescription dose received was 99.7 Gy (range: 61.7–120 Gy). Treatment characteristics at reirradiation are presented in **Table 2**. The median interval between completion of initial RT and the beginning of reirradiation was 25.9 months (range: 1.6–214.2 months). Seven patients (3.0%) were treated with reirradiation within 6 months of completing initial RT, and 32 patients (13.9%) were treated within 12 months. Of those with evaluable initial and reirradiation treatment plans, the median cumulative maximum doses to the brainstem and the optic apparatus (optic chiasm and nerves) were 77.9 Gy (range: 4.6–146.0 Gy) and 55.1 Gy (range: 3.3–106.3 Gy), respectively. Seventy patients had plans available for evaluation by coregistration; for these, the median cumulative maximum doses to the brainstem and the optic apparatus were 76.6 Gy (range: 6.3–110.8 Gy) and 54.5 Gy (range: 4.2–106.3 Gy), respectively. Most patient had direct overlap of treatment fields or type 1 reirradiation (82.2%, 189/230). Eight patients had no overlap of treatment fields (3.5%). Treatment field overlap could not be retrospectively determined in 14.3% of patients (33/230).

**Table 2.** Treatment Information

	All Patients				IDH Wildtype Subanalysis			
	Median	Min		Max	Median	Min		Max
Reirradiation dose received (Gy)	41.4	1.8		60	45	30.6		60
Reirradiation volume (cm^3^)	199.6	15.9		900.6	183.0	15.9		824.9
Cumulative maximum brainstem dose (Gy)								
All evaluable plans^a^ (Gy)	77.9	4.6		146.0	76.3	4.6		146.0
Composite only^b^ (Gy)	76.6	6.3		110.8	70.5	6.3		102.2
Cumulative maximum optic structure dose (Gy)								
All evaluable plans^a^ (Gy)	55.1	3.3		106.3	54.0	4.2		103.2
Composite only^b^ (Gy)	54.5	4.2		106.3	51.6	4.2		81.9
Time from initial to repeat radiation (mo)	25.9	1.6		214.2	20.2	1.6		214.2

	Number		%		Number		%	
Concurrent Systemic therapy								
Temozolomide	147		63.9		68		68.7	
Bevacizumab	20		8.7		8		8.1	
Temozolomide + bevacizumab	16		7.0		6		6.1	
Other	2		0.9		2		2.0	
None	45		19.6		15		15.2	
Surgery								
GTR	47		20.4		16		16.2	
STR	64		27.8		30		30.3	
Biopsy only	3		1.3		1		1.0	
Surgery, margin status unknown	22		9.6		9		9.1	
None	93		40.4		43		43.4	
Unknown	1		0.4		0		0	
Technique								
IMRT	213		92.6		91		91.9	
Proton Beam Therapy	16		7.0		7		7.1	
3D-CRT	1		0.4		1		1.0	
Reirradiation Type								
Type 1	189		82.2		82		82.8	
Type 2	8		3.5		6		6.1	
Unknown	33		14.3		11		11.1	
Completed re-RT	214		93.0		N/A			

Abbreviations: Gy = Gray; cm^3^ = centimeter cubed; GTR = gross total resection; STR = subtotal resection; IMRT = intensity modulated radiotherapy; 3D-CRT = three-dimensional conformal radiotherapy; RT = radiotherapy; N/A = not applicable.

Cumulative maximum brainstem and optic structure doses are reported for:.

^a^All patients with plan information from initial and reirradiation treatments (summation of maximum point doses anywhere in the structure when composite plan fusion not available) (n = 145).

^b^Patients with composite plan fusions only (n = 70).

Sixty percent of patients had surgical management at time of recurrence before reirradiation (20.4% gross total resection [GTR] [47/230], 27.8% subtotal resection [64/230], 9.6% margin status unknown [22/230], 1.3% biopsy only [3/230). Concurrent TMZ was given to 63.9% of patients (147/230), BEV to 8.7% (20/230), and TMZ + BEV to 7.0% (16/230), and no systemic therapy was used in 19.6% of patients (45/230). Ninety-three percent of patients (214/230) completed their planned reirradiation course (**Table 2**), which was not affected by the use of concurrent systemic therapy (*P* > .05 on chi square test).

### Treatment Toxicity

Minimal neurotoxicity was observed in this cohort treated with reirradiation. Acute grade 3+ toxicity was seen in 9.6% of patients (22/230), and 70.4% (162/230) experienced grade 2 toxicity, primarily from the short-term requirement of steroids during or within 3 months of reirradiation. Generally, concurrent use of BEV was reserved for highest risk or most symptomatic patients with the goal of controlling edema. Concurrent BEV was not associated with less acute toxicity. Late grade 3+ toxicities were seen in 6.5% (15/230): 12 cases included focal motor weakness, 3 cases included aphasia and/or dysarthria, 1 case included hemineglect, and 1 case included urinary incontinence. Radiation necrosis was reported in 7.8% (18/230) of all patients and 9.4% (18/192) of cases where post-reirradiation imaging were obtained (**Table 3**). The median time to radiation necrosis was 2.1 months from reirradiation start (range: 0.9–6.8 months). Of those diagnosed with radiation necrosis, 83.3% (15/18) were diagnosed by imaging alone, while 16.7% (3/18) were confirmed by surgical pathology. Regarding radiation necrosis grading, 5.7% (1/18), 72.2% (13/18), and 22.2% (4/18) were grade 1, grade 2, and grade 3 toxicity, respectively. No episodes of radiation necrosis involving the brainstem were observed. Steroids were required within three months in 80.0% (184/230) of all cases, and 82.1% (184/224) of cases that could be accurately evaluated for this variable, retrospectively. With median cumulative maximum doses to the brainstem and optic structures of 77.9 Gy (range: 4.6–146.0 Gy) and 55.1 Gy (range: 3.3–106.3 Gy), respectively, there were no identified symptomatic injuries to these structures. Late grade 3+ RTOG CNS toxicity, radiation necrosis, and short-term steroid requirement were not significantly affected by reirradiation volume, time duration between initial and reirradiation ≥ 24 months, or reirradiation dose received ≥41.4 Gy (*P* > .05 on chi square test or Fisher’s exact test).

**Table 3.** Acute and Late Treatment-related Toxicity

Neurotoxicity	Number	%
RTOG acute CNS morbidity		
Grade 0	15	6.5
Grade 1	21	9.1
Grade 2	162	70.4
Grade 3	21	9.1
Grade 4	1	0.4
Unknown	10	4.3
RTOG late CNS morbidity		
Grade 0	53	23.0
Grade 1	47	20.4
Grade 2	49	21.3
Grade 3	15	6.5
Unknown	14	6.1
N/A	52	22.6
Radiation necrosis		
Yes	18	7.8
No	174	75.7
Unknkown	38	16.5
Hematologic toxicity		
Lymphopenia^a^		
Grade 0	52	30.4
Grade 1	35	20.5
Grade 2	42	24.6
Grade 3	36	21.1
Grade 4	6	3.5

Abbreviations: RTOG = Radiation Therapy Oncology Group; CNS = central nervous system; N/A = not applicable.

^a^Lymphopenia defined by Common Terminology Criteria for Adverse Events version 5.0^9^ (*n* = 171).

Concurrent systemic therapy was utilized with reirradiation in 80.4% of cases. During or within 1 month of reirradiation: grade 3+ anemia occurred in 0.5% of patients (1/185); grade 3+ leukopenia occurred in 2.2% (4/185); grade 3+ neutropenia occurred in 2.9% (5/171); and grade 3+ thrombocytopenia occurred in 3.8 (7/185)%. Notably, grade 3+ lymphopenia occurred in 24.6% of cases (42/171) (**Table 3**).

### Survival Outcomes

**Figure 1.**
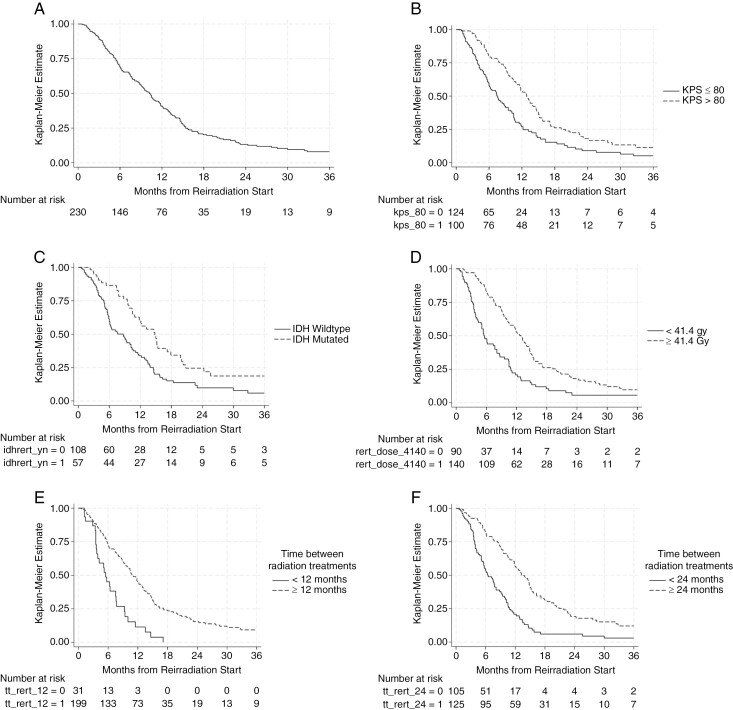
Overall survival from start of reirradiation for (A) all patients, (B) Karnofsky Performance Status ≤80 versus >80; (C) isocitrate dehydrogenase (IDH) wildtype versus mutated, (D) those with reirradiation to <41.4 Gy versus ≥ 41.4 Gy, (E) those with < 12 months versus ≥ 12 months between radiation treatments, (F) those with < 24 months versus ≥ 24 months between radiation treatments.

Median OS from the start of reirradiation for all patients was 10.2 months (95% CI 8.8–11.7 months) (**Figure 1**). Median OS from the start of reirradiation for those treated within 6-, 12-, and 24 months of initial radiotherapy were 5.2 months (1.4–5.7 months), 5.5 months (3.6–7.6 months), and 6.4 months (5.5–8.1 months), respectively. The median OS for patients treated with reirradiation ≥ 24 months from initial radiotherapy was 13.2 months (11.8–15.0 months). On univariate analysis, improved survival was seen in those with higher KPS, lower grade of initial diagnosis, IDH-mutated disease, longer interval between initial and reirradiation, reirradiation at first recurrence, reirradiation dose received ≥ 41.4 Gy, smaller target volume, and absence of grade 3+ lymphopenia (**Table 4**). On multivariate analysis, higher KPS, longer interval between radiotherapy sessions, radiotherapy at first recurrence, and reirradiation dose ≥ 41.4 Gy remained significantly associated with improved survival. Reirradiation target volume was no longer associated with OS (**Table 4**).

**Table 4.** Overall Survival from Start of Reirradiation

		Univariate		Multivariate	
Variable	Median OS months (95% CI)	HR (95% CI)	*P*-value	HR (95% CI)	*P*-value
Age, y					
≤50	11.8 (10.2–13.9)	0.76 (0.57–1.02)	.07		
> 50	8.1 (6.2–10.2)	Ref			
KPS					
≤ 80	7.5 (6.0–9.5)	1.75 (1.30–2.36)	<.001	1.59 (1.13–2.22)	.007
> 80	12.9 (10.9–14.6)	Ref		Ref	
Initial grade					
1-2	11.6 (7.5–15.2)	0.58 (0.39–0.88)	.006		
3	12.2 (9.6–13.9)	0.64 (0.45–0.90)	.01		
4	8.1 (6.4–10.2)	Ref			
IDH					
Wildtype	8.7 (6.0–10.2)	Ref			
Mutated	14.8 (10.5–17.9)	0.51 (0.34–0.75)	.001		
MGMT promoter					
Nonmethylated	7.7 (5.9–10.2)	Ref			
Methylated	11.8 (7.8–14.0)	0.71 (0.50–1.02	.06		
Time between radiation treatments, mo					
< 24 mo	6.4 (5.5–8.1)	Ref		Ref	
≥ 24 mo	13.2 (11.8–15.0)	0.42 (0.31–0.57)	<.001	0.47 (0.34–0.65)	<.001
Reirradiation at first recurrence					
First recurrence	11.1 (9.6–13.5)	0.67 (0.50–0.90)	.008	0.63 (0.46–0.88)	.006
Subsequent recurrence	7.8 (6.0–10.2)	Ref		Ref	
Surgery prior to reirradiation					
GTR	12.8 (8.1–15.4)	Ref			
STR	11.5 (7.5–13.4)	1.24 (0.81–1.90)	.3		
None	8.8 (6.3–10.2)	1.42 (0.94–2.12)	.09		
Concurrent systemic therapy					
None	10.0 (5.4–15.4)	Ref			
TMZ	11.5 (9.8–12.8)	0.93 (0.62–1.38)	.7		
BEV	7.7 (3.6–8.4)	1.67 (0.93–2.99)	.09		
TMZ+BEV	5.9 (2.9–9.5)	2.07 (1.11–3.85)	.02		
Reirradiation dose received					
< 41.4 Gy	5.6 (4.3–8.1)	Ref		Ref	
≥ 41.4 Gy	12.9 (10.6–14.5)	0.48 (0.35–0.64)	<.001	0.55 (0.40–0.76)	<.001
Cumulative max brainstem dose					
< 92 Gy	9.8 (7.8–11.6)	Ref			
≥ 92 Gy	7.5 (3.9–13.5)	1.15 (0.75–1.76)	.5		
Reirradiation volume					
< 200 cm^3^	11.6 (9.5–12.9)	Ref		Ref	
≥ 200 cm^3^	7.7 (5.9–10.4)	1.43 (1.06–1.91)	.02	1.05 (0.75–1.45)	.8
Lymphopenia^a^					
Grade 0–2	11.8 (10.5–13.4)	Ref			
Grade 3+	7.2 (5.5–9.5)	1.77 (1.19–2.63)	.005		

Abbreviations: OS = overall survival; CI = confidence interval; HR = hazard ratio; y = year; Ref = reference. KPS = Karnofsky performance status; IDH = isocitrate dehydrogenase; MGMT = O^6^-methylguanine DNA methyltransferase; mo = months; GTR = gross total resection; STR = subtotal resection; TMZ = temozolomide; BEV = bevacizumab; Gy = gray; cm^3^ = centimeter cubed.

^a^Lymphopenia defined by Common Terminology Criteria for Adverse Events version 5.0.^9^.

Median OS from the start of reirradiation for patients with IDH wildtype disease was 8.7 months (95% CI 6.0–10.2 mo) (**Table 4**, **Figure 1**). A subanalysis was completed of patients with IDH wildtype disease who completed reirradiation. On univariate analysis of these patients, longer interval between radiotherapy sessions and reirradiation dose received ≥ 41.4 Gy were associated with improved survival. Age, higher KPS, MGMT promoter methylation, reirradiation at first recurrence, and larger reirradiation volume were not associated with OS when limited to patients with IDH wildtype disease. On multivariate analysis, both longer interval between radiotherapy sessions and reirradiation dose received ≥ 41.4 Gy remained significant predictors of OS (**Table 5**).

**Table 5.** Overall Survival from Start of Reirradiation in Patients with IDH Wildtype Glioblastoma who Completed Reirradiation

		Univariate		Multivariate	
Variables	Median OS months (95% CI)	HR (95% CI)	*P*-value	HR (95% CI)	*P*-value
Age, y					
≤50	10.4 (5.9–13.9)	0.81 (0.49–1.32)	.4		
> 50	8.7 (6.1–10.6)	Ref			
KPS					
≤ 80	6.3 (5.6–10.0)	1.28 (0.81–2.03)	.3		
> 80	11.1 (9.0–13.5)	Ref			
MGMT promoter					
Nonmethylated	6.5 (5.6–10.2)	Ref			
Methylated	10.6 (6.1–14.6)	0.67 (0.42–1.07)	.1		
Time between radiation treatments, mo					
< 12 mo	6.4 (3.9–9.4)	Ref		Ref	
≥ 12 mo	10.2 (6.2–13.1)	0.61 (0.38–0.97)	.03	0.46 (0.27–0.81)	.006
Reirradiation at first recurrence					
First recurrence	9.4 (6.3–13.1)	0.69 (0.44–1.09)	.1		
Subsequent recurrence	6.5 (5.1–10.2)	Ref			
Reirradiation dose received					
< 41.4 Gy	5.4 (4.1–10.4)	Ref		Ref	
≥ 41.4 Gy	10.2 (7.6–13.4)	0.56 (0.35–0.88)	.01	0.50 (0.31–0.80)	.004
Reirradiation volume					
< 200 cm^3^	10.2 (7.5–13.5)	Ref			
≥ 200 cm^3^	6.1 (5.4–10.2)	1.53 (0.98–2.40)	.06		

Abbreviations: CI = confidence interval; HR = hazard ratio; y = year; Ref = reference; KPS = Karnofsky performance status; MGMT = O^6^-methylguanine DNA methyltransferase; mo = months; Gy = gray; cm^3^ = centimeter cubed.

## Discussion

Reirradiation of HGG using doses of 41.4–45 Gy (or 54 Gy in cases of no treatment field overlap), along with the use of concurrent daily TMZ, appears to be a safe salvage option following a recurrence event. The current study supports the use of reirradiation doses ≥ 41.4 Gy for HGG as well as treatment of large target volumes ≥ 200 cm^3^ as neither were associated with increased risk of treatment-related toxicity, and these may improve OS. Median survival from reirradiation for the entire cohort was 10.2 months (95% CI 8.8–11.7 months). On subanalysis of those with IDH wildtype disease, higher reirradiation dose continued to be associated with improved survival (HR 0.50, 95% CI 0.31–0.80).

Our group previously identified variables that may predict which patients benefit from salvage reirradiation following recurrent HGG.^3^ Within our prior study that evaluated patients with recurrent HGG treated with fractionated radiotherapy between 2007 and 2016, time between radiation sessions > 24 months, reirradiation dose > 41.4 Gy, and extent of surgical resection before reirradiation predicted improved survival. The current study updates these findings by evaluating more recently treated patients between 2007 and 2022 and analyzes those with recurrent glioblastoma as defined by the World Health Organization Central Nervous System 2021 Classification.^10^ In the evaluation of all patients with recurrent grade 3–4 glioma, KPS > 80, time between radiation sessions ≥ 24 months, reirradiation at first recurrence, and reirradiation dose ≥ 41.4 Gy were associated with improved OS on multivariate analysis. MGMT promoter methylated disease had a hazard ratio of 0.71 (95% CI 0.50–1.02, *P* = .06) on univariate analysis but power was limited in that MGMT status was unknown for 32.2% of patients, especially in the earlier years when determination was not standard. Glioma grade at the initial diagnosis was a significant predictor on univariate analysis, but this was withheld from multivariate analysis due to collinearity with time between radiation sessions. IDH wildtype disease and grade 3+ lymphopenia were also significant predictors on univariate analyses. These variables were available in a subset of patients and, thus, were not included in the multivariate analysis. Instead, a subanalysis of IDH wildtype disease was completed which revealed similar results.

The low incidence of normal tissue injury, including brain necrosis, brainstem functional injury, and significant optic nerve/chiasm injury, suggest that fractionated reirradiation is safe. Notably, BEV which is hypothesized to improve safety and was concurrently administered used in the only large prospective study of HGG reirradiation (RTOG 1205),^11^ was utilized in only 15.7% of patients in the current study. Higher reirradiation dose was associated with improved survival, including for patients with larger target volumes. Clinicians often consider several factors when assessing a patient’s risk of radiation necrosis including dose fractionation, treated volume size, systemic therapy use, and the interval between radiation sessions.^1,12^ Nevertheless, brain tissue dose constraints remain unclear within the reirradiation setting.^1,13^ The QUANTEC project predicted a 5% and 10% risk of symptomatic radiation necrosis occurrence at 72 Gy and 90 Gy, respectively in 2-Gy equivalent dosing (alpha/beta ratio of 3).^14^ Practice patterns of cranial reirradiation vary widely, with many exceeding such dose constraints. A recent review of reirradiation for HGG observed a median cumulative EQD2 around 100–110 Gy to be associated with a < 10% risk of radiation necrosis. The rate of radiation necrosis in the current study was 9.4%, while the median cumulative EQD2 was 99.7 Gy (range: 61.7–120 Gy). The median target volume size of those treated with conventionally fractionated reirradiation in the aforementioned review article ranged from 49 to 202 cm.^1,3^ The median treated volume of the current series is larger than most at 200 cm^3^. That said, there was no association between radiation necrosis and PTV size in the current study. There was also no significant differences in radiation necrosis rates when stratified by use of concurrent systemic therapy or the time interval between radiation sessions. In selected patients, higher reirradiation doses may be safely delivered to large target volumes. Although RTOG 1205 included BEV for all reirradiated patients based on the hypothesis of improving tumor outcome and reducing risks of repeat radiation,^11^ we recommend use only in selected circumstances as we show that treatment without this agent is appropriately safe. Furthermore, randomized data have since shown no benefit when BEV is given concurrently in the initial therapy of GBM.^15^

These data suggest that higher than expected cumulative dose to brainstem and optic nerves/chiasm may be tolerable. Predictive modeling of dose-volume data has identified suggested maximum doses for a single first course of radiation to be 55 Gy and 54 Gy (59 Gy < 10 cc) given in 1.8–2 Gy fractions for the optic apparatus and the brainstem, respectively.^1,16^ Cumulative maximum doses in the current series were determined by co-registered plans for 70 patients (when available) or by the addition of maximum doses to contours. The median cumulative maximum dose delivered to the optic apparatus and the brainstem were 55 Gy (range: 3–106 Gy) and 78 Gy (range: 5–146 Gy), respectively. No episodes of symptomatic injury to these structures were identified. In addition, a higher maximum dose delivered to the brainstem within the range utilized in this cohort was not associated with radiation necrosis. Niyazi et al. similarly saw no relevant long-term toxicity in such structures for patients with malignant gliomas treated with reirradiation.^17^ Within their series of 58 patients who received repeat radiotherapy to 36 Gy in 18 fractions, the maximum cumulative EQD2 was 80.3 Gy, 79.4 Gy, and 95.2 Gy for the optic chiasm, optic nerves, and brainstem, respectively. These data highlight the need to rethink dose constraints in the reirradiation setting or suggest the consideration of tissue recovery between initial and reirradiation.^13,18^

Many studies have evaluated the safety and efficacy of systemic therapy with or without reirradiation in HGG. Within our study population, there were no associations between concurrent systemic therapy use and toxicity rates although 70.9% received concurrent TMZ with reirradiation. Less than 20% of patients received BEV, with or without TMZ. The retrospective nature of this study and patient selection limits other conclusions that may be made regarding systemic therapy use with reirradiation. MGMT promoter methylation status which is associated with better response to TMZ was only known for a subset of cases.

There are limited data regarding the efficacy of reirradiation specific for those with IDH wildtype confirmed glioblastoma. Within our cohort, the median OS for those with IDH wildtype disease was 8.7 months from reirradiation which was lower than those with IDH mutated tumors. Christ et al. recently described a single-institution experience of reirradiation for recurrent IDH wildtype glioblastoma which included 129 eligible patients treated either with SRS/SRT in 1–5 fractions (29%) or fractionated radiotherapy (54%).^19^ In this selected population, the median PTV size was 39 cm^3^. The median OS in this study was 7.3 months from reirradiation, and higher KPS was associated with improved OS on multivariate analysis. Ehret et al. evaluated outcomes of 88 patients with IDH wildtype glioblastoma and only included patients treated with hypofractionated or conventionally fractionated reirradiation. The median PTV size was 98 cm^3^. The median OS in their study was 8.0 months, and GTR (versus biopsy or no resection) was associated with improved OS on multivariate analysis. Within our cohort, the median OS for those with IDH wildtype disease was comparable to other studies at 8.7 months from reirradiation (95% CI 6.0–10.2). The median PTV size for this subanalysis cohort was larger than others at 183 cm^3^. On multivariate analysis of those with IDH wildtype disease, the time between radiation sessions ≥ 12 months and reirradiation dose ≥ 41.4 Gy were associated with improved survival.

This study has several limitations that warrant consideration. First, this is a retrospective, single-institution review, which may be vulnerable to selection bias. Second, treatment for each patient and dose limits were determined by the individual treating physicians. Though the variability in practice patterns across a large department may help in the efficacy and toxicity evaluations of different fractionation schemes, there may also be differences in CTV and PTV expansions and other biases as there is no consensus standard. Third, we recognize that selection criteria played a major role in those who received no concurrent systemic therapy and those who received either BEV or TMZ+BEV, and thus, any comparisons among these are difficult to make. Lastly, these patients were treated over a 15-year span, during which practice patterns and alternative salvage therapies may have changed. Also, over this time, molecular profiling of tumors significantly changed. Earlier in this cohort, IDH mutations were often confirmed by immunohistochemical (IHC) staining, while more recently, IDH mutations were confirmed by next-generation sequencing. Thus, some earlier patients identified as having IDH wildtype disease may have harbored IDH mutations not identified by IHC. In addition, necrosis was generally diagnosed based on imaging review rather than pathologic confirmation.

## Conclusion

Reirradiation, generally to a total dose of 41.4–45 Gy at 1.8 Gy per fraction with concurrent temozolomide, is an appropriate treatment for recurrent HGG including large tumors, those near critical structures, and those treated after 6 months from their initial RT resulting in a median survival of 10.2 months. Favorable prognostic factors included higher performance status, longer interval between radiotherapy sessions, reirradiation at first recurrence, and reirradiation dose ≥ 41.4 Gy. The acceptable incidence of radiation necrosis and absence of optic nerve and brainstem injury suggest that exploration of higher reirradiation dose can be considered. This treatment is appropriately safe without the use of concurrent bevacizumab. These findings may be used to inform patient selection in future prospective study of appropriate reirradiation fractionation schemes, and guide design of studies evaluating both concurrent standard and novel systemic therapies.

## Data Availability

The datasets used and/or analyzed during the current study are available from the corresponding author on reasonable request.

## References

[1] Minniti G , Niyazi M, Alongi F, Navarria P, Belka C. Current status and recent advances in reirradiation of glioblastoma. Radiat Oncol. 2021;16(1):36. 33602305 10.1186/s13014-021-01767-9 PMC7890828

[2] Kazmi F , Soon YY, Leong YH, Koh WY, Vellayappan B. Re-irradiation for recurrent glioblastoma (GBM): a systematic review and meta-analysis. J Neurooncol. 2019;142(1):79–90. 30523605 10.1007/s11060-018-03064-0

[3] Shen CJ , Kummerlowe MN, Redmond KJ, et al Re-irradiation for malignant glioma: Toward patient selection and defining treatment parameters for salvage. Adv Radiat Oncol. 2018;3(4):582–590. 30370358 10.1016/j.adro.2018.06.005 PMC6200913

[4] Navarria P , Minniti G, Clerici E, et al Re-irradiation for recurrent glioma: outcome evaluation, toxicity and prognostic factors assessment. A multicenter study of the Radiation Oncology Italian Association (AIRO). J Neurooncol. 2019;142(1):59–67. 30515706 10.1007/s11060-018-03059-x

[5] Marwah R , Xing D, Squire T, et al Reirradiation versus systemic therapy versus combination therapy for recurrent high-grade glioma: a systematic review and meta-analysis of survival and toxicity. J Neurooncol. 2023;164(3):505–524. 37733174 10.1007/s11060-023-04441-0 PMC10589175

[6] Zarabi H , Helis CA, Russell G, et al Multi-institutional report of re-irradiation for recurrent high-grade glioma. Int J Radiation Oncol*Biol*Phys. 2023;117(2):S85–S86.

[7] Andratschke N , Willmann J, Appelt AL, et al European society for radiotherapy and oncology and European Organisation for research and treatment of cancer consensus on re-irradiation: definition, reporting, and clinical decision making. Lancet Oncol. 2022;23(10):e469–e478. 36174633 10.1016/S1470-2045(22)00447-8

[8] Cox JD , Stetz J, Pajak TF. Toxicity criteria of the Radiation Therapy Oncology Group (RTOG) and the European Organization for Research and Treatment of Cancer (EORTC). Int J Radiat Oncol Biol Phys. 1995;31(5):1341–1346. 7713792 10.1016/0360-3016(95)00060-C

[9] Protocol Development. https://ctep.cancer.gov/protocoldevelopment/electronic_applications/ctc.htm. Accessed March 31, 2024.

[10] Louis DN , Perry A, Wesseling P, et al The 2021 WHO classification of tumors of the central nervous system: a summary. Neuro Oncol. 2021;23(8):1231–1251. 34185076 10.1093/neuonc/noab106 PMC8328013

[11] Tsien CI , Pugh SL, Dicker AP, et al NRG Oncology/RTOG1205: a randomized phase II trial of concurrent bevacizumab and reirradiation versus bevacizumab alone as treatment for recurrent glioblastoma. J Clin Oncol. 2023;41(6):1285–1295. 36260832 10.1200/JCO.22.00164 PMC9940937

[12] Winter SF , Loebel F, Loeffler J, et al Treatment-induced brain tissue necrosis: a clinical challenge in neuro-oncology. Neuro Oncol. 2019;21(9):1118–1130. 30828724 10.1093/neuonc/noz048 PMC7594558

[13] Mayer R , Sminia P. Reirradiation tolerance of the human brain. Int J Radiat Oncol Biol Phys. 2008;70(5):1350–1360. 18037587 10.1016/j.ijrobp.2007.08.015

[14] Lawrence YR , Li XA, el Naqa I, et al Radiation dose-volume effects in the brain. Int J Radiat Oncol Biol Phys. 2010;76(3 Suppl):S20–S27. 20171513 10.1016/j.ijrobp.2009.02.091 PMC3554255

[15] Gilbert MR , Dignam JJ, Armstrong TS, et al A randomized trial of bevacizumab for newly diagnosed glioblastoma. N Engl J Med. 2014;370(8):699–708. 24552317 10.1056/NEJMoa1308573 PMC4201043

[16] Mayo C , Martel MK, Marks LB, et al Radiation dose-volume effects of optic nerves and chiasm. Int J Radiat Oncol Biol Phys. 2010;76(3 Suppl):S28–S35. 20171514 10.1016/j.ijrobp.2009.07.1753

[17] Niyazi M , Karin I, Söhn M, et al Analysis of equivalent uniform dose (EUD) and conventional radiation treatment parameters after primary and re-irradiation of malignant glioma. Radiat Oncol. 2013;8:287. 24330746 10.1186/1748-717X-8-287 PMC4029146

[18] Kirkpatrick JP , van der Kogel AJ, Schultheiss TE. Radiation dose-volume effects in the spinal cord. Int J Radiat Oncol Biol Phys. 2010;76(3 Suppl):S42–S49. 20171517 10.1016/j.ijrobp.2009.04.095

[19] Christ SM , Youssef G, Tanguturi SK, et al Re-irradiation of recurrent IDH-wildtype glioblastoma in the bevacizumab and immunotherapy era: Target delineation, outcomes and patterns of recurrence. Clin Transl Radiat Oncol. 2024;44:100697. 38046107 10.1016/j.ctro.2023.100697 PMC10689476

